# Parents' dilemma: A therapeutic decision for children with spinal muscular atrophy (SMA) type 1

**DOI:** 10.3389/fped.2022.1062390

**Published:** 2022-12-21

**Authors:** Sophie Boursange, Marco Araneda, Caroline Stalens, Isabelle Desguerre, Christine Barnerias, Marie-Christine Nougues, Arnaud Isapof, Susana Quijano-Roy, Nadia Blu Genestine, Laetitia Ouillade, Maripaz Martinez Jalilie, Claudia Castiglioni, Odile Boespflug-Tanguy, Marcela Gargiulo, The SMAPAR Study Group

**Affiliations:** ^1^ Université Paris Cité, Laboratoire de Psychologie Clinique, Psychopathologie, Psychanalyse, Boulogne-Billancourt, France; ^2^ Université Paris Cité, Ecole Doctorale ED-261, “Cognition, Conduct and Human Behavior”, Boulogne-Billancourt, France; ^3^ Institut de Myologie, Hôpital de la Pitié-Salpêtrière, APHP-Paris, France; ^4^ Université Paris Cité, CRPMS, F-75013 Paris, France; ^5^ French Association against Myopathies (AFM), Public Health and Medical Research Department, Evry, France; ^6^ Centre de Référence des Maladies Neuromusculaires, Centre Nord-Est-Ile de France, Réseau National des Maladies Neuromusculaires, FILNEMUS, France; ^7^ European Reference Center Network (Euro-NMD ERN), Paris, France; ^8^ Pediatric Neurology Department, AP-HP Hôpital Necker Enfants Malades, Paris, France; ^9^ Department of Paediatric Neurology, Armand Trousseau Hospital, Assistance Publique-Hôpitaux de Paris, France; ^10^ APHP Université Paris Saclay, Neuromuscular Unit Pediatric Neurology and ICU Department, Raymond Poincarré Hospital, Garches, France; ^11^ ECLAS association, Together against SMA type 1, Saint Denis Le Gast, France; ^12^ SMA Interest Group – AFM Téléthon, Evry, France; ^13^ Unidad de Investigación, Escuela de Medicina, Universidad Finis Terrae, Santiago, Chile; ^14^ Neuropediatric Department, Las Condes Clinic, Santiago Chile, Chile; ^15^ I-Motion, Institute of Myology, Armand Trousseau Hospital, Assistance Publique-Hôpitaux de Paris, France; ^16^ Université Paris Cité, UMR 1141, Paris, France

**Keywords:** dilemmatic decisions, palliative care, parents, caregivers, spinal amyotrophy type 1, SMAPAR Study, burden, innovative therapies

## Abstract

**Background:**

SMA type 1 is a severe neurodegenerative disorder that, in the absence of curative treatment, leads to death before 1 year of age without ventilatory support. Three innovative therapies are available to increase life expectancy.

**Purpose:**

(i) To increase knowledge about parents' experiences with their decision to have opted for an innovative therapy; (ii) to assess the middle-term psychological consequences in the parents' lives.

**Methods:**

We used an in-depth interview; a self-administrated questionnaire and self-report scales (BDI-II, STAI-Y, PSI-SF, SOC-13, PBA, DAS 16 and FICD). We compared parents hesitant before the decision to parents who were not-hesitant and the group of parents whose child was treated with gene therapy (GT) to parents whose child received another innovative therapy.

**Main results:**

We included *n* = 18 parents of 13 children. Parent's mean age was 34.7 (±5.2), child's average age was 44.3 months (±38.0). Retrospectively, most parents felt involved by doctors in decision-making on treatment, they felt their point of view was considered and were satisfied with the effects of the treatment. The group of parents “non-hesitant” was more depressed (*p* < 0.001), more anxious (*p* = 0.022) and had higher parental stress (*p* = 0.026) than the group of “hesitant” parents; the group of “GT-treated” parents was more depressed (*p* = 0.036) than the group of parents with “other therapy”. Qualitative data highlights revealed: the need to save the child's life at all costs; the fear of coping with end of life and palliative care, the high value of perceived physician confidence in the treatment, the hope that the child will acquire autonomy or be cured. At the time of the decision, no parents felt they fully understood all of the issues regarding therapy and the disease.

**Conclusion:**

Hesitating before making a decision did not predispose parents to depression and anxiety. The narratives suggest that the parents faced a dilemma regarding their child's health in an urgent context. The decision was not final, and parents will continue to think about it throughout the care process.

## Introduction

Spinal muscular atrophy (SMA) is a degenerative disease of the motor neurons that, emerge from the spinal cord and brainstem. It is due to an alteration of the survival motor neuron 1 (*SMN1*) gene on the two alleles of maternal and paternal origin in the genome of the affected child. Parents transmitting the anomaly never develop the disease, as it is an autosomal recessive disease. It is the leading cause of pediatric neurodegenerative genetic disease, with an incidence in France of 120–150 births per year (15–19 new cases/100.000 births) and the second most common genetic pediatric disease after cystic fibrosis (1–3). These numbers are likely overestimated as only 35–40 new cases per year have been evaluated during national multidisciplinary consensus meetings (MCM) of reference centers for rare neuromuscular diseases (FILNEMUS) as of June 2018. SMA type 1 is the most severe and common form (50%–60%) of SMA. It appears within the first 6 months of life and is characterized by significant muscle weakness and very little motor acquisition (the independent sitting position is not acquired). Bulbar functions (swallowing disorders) and respiratory (ventilatory disorders) functions are also impaired, the latter being a key factor in the severity of the disease (1, 2, 4). In France, the various natural histories come from the follow-up of nearly 200 patients over 20 years (1999–2016). The patients received active palliative care but no long-term ventilation (non-invasive ventilation or tracheotomy). They showed a rapid evolution with a median age of diagnosis at 3 months (range 0.6–10.4) and a median age of death at 6 months (range 1–27) (5). A subgroup of children (type 1c) was defined by onset between three and 6 months (acquired head posture) usually with longer survival but a very severe disability (2). In other hand, *SMN2* gene has been found to modulate the age of onset and severity of loss of function of the *SMN1* gene (6). The first clinical trials began in 2008 and led to the approval for the first innovative therapy in the USA in 2016 (7), then in Europe in 2017 (8).

Currently, three innovative therapies^1^ can increase life expectancy and improve motor and respiratory functions. Clinical studies show greater and faster efficacy when treatments are initiated early and ideally at a pre-symptomatic stage (9). Unlike in other countries, systematic neonatal screening is not yet available in France, which limits the possibility of treatment immediately from birth (6). A pilot project, DEPISMA, is being tested in two French regions. While these therapies change the natural course of the disease and bring new perspectives for patients and their families, many uncertainties remain (10) and families and medical teams navigate between “isolated islands of certainty in an ocean of uncertainty” (11).

In France, the therapeutic management of children with SMA needs to be ratified during MCM of reference centers for rare neuromuscular diseases (FILNEMUS) (12). Doctors discuss treatment indications based on the clinical (motor, bulbar and respiratory functions), the child's genetic profile and the available scientific data. They are thus faced with the ethical dilemma of choosing between palliative support through the end of life, or implementing an innovative therapy. The decision is most often broached with parents during the diagnostic confirmation visit and the final decision is an emergency. When the medical team advises palliative care, but the parents want an innovative therapy, they may experience the decision as an imposed sentence and often seek a second opinion. When the MCM proposes innovative therapy, the final decision about whether to treat the child is always up to the parents. They may sometimes interpret this proposal as a solution repairing the shock of the announcement of the child's illness, particularly when the SMA is severe (13). After the final decision, therapies are most of the time initiated 2 weeks after the genetic diagnosis has been confirmed.

In this urgent context, parents of children with SMA type 1 are faced with a dilemma: the decision to either consent to innovative therapy or to pursue palliative care and accompany their child through the end of life.

The aim of this study is to describe how parents experience their prior decision regarding an innovative therapy for their child with SMA type 1. This is a psychoanalytically oriented clinical psychology research based on a triangulation of quantitative and qualitative data.

This article presents the results of the subgroup of parents of children with SMA type 1 of the French cohort, of the SMAPAR study: a large Franco-Chilean study looking at the experience of parents of children (aged 1–18) with SMA type 1, 2 or 3 and the feeling of parental overload.

Objectives: (i) to increase knowledge about parents' experiences on their prior decision for an innovative therapy for their child with SMA type 1; (ii) to assess the middle-term psychological consequences in their lives.

## Patients and methods

The inclusion of parents in the SMAPAR study took place in France between January 1, 2021 and September 30, 2021, within specialized neuropediatric departments (FILNEMUS) who chose to participate and through patient associations: French Association against Myopathies (AFM-Telethon) and Together Against SMA type 1 association (ECLAS). The parents who participated did so voluntarily and both parents within a couple could participate independently.

We used a mixed methodology with both qualitative tools (in-depth semi-structured interviews) and quantitative tools (self-questionnaire developed by members of the Steering Comitee of the SMAPAR study and validated in conjunction with members of patient associations and 7 self-questionnaires evaluating the medium-term psychological consequences on the parents). Complete methodology of the SMAPAR study is available in the Supplementary Material. Mixed methods propose to combine, in a more or less intimate way, quantitative and qualitative methods in order to produce results that combine credibility and meaning (14).

The study complies with MR-004 reference methodology that provides a framework for the processing of personal data for the purpose of studies, evaluations or research not involving the human person as studies that do not meet the definition of research involving the human person. It received approval from the Ethics Committee (No: #00011928, December 15, 2020) including the use of reported speech verbatim in publications. All participants signed written statements of non-opposition.

To meet the objectives of this study: we identified the variables related to therapeutic decision-making, mental health, and the impact of the disease on daily life and family in the catalog of data from the SMAPAR study:

(1) Variables from the in-house self-questionnaire: the self-questionnaire consisted of open and closed multiple-choice questions and visual analog scales (VAS) that ranged from 0 (lowest agreement or satisfaction score) to 100 (highest agreement or satisfaction score).

We isolated the following variables:

- *Parents*: sex, age, marital status (multiple choice), parental status (primiparous Y/N), economic status (multiple choice), professional status (multiple choice), social assistance and coverage status (multiple choice), perception of overall health (VAS) and impact of the disease on the mental and physical health (Y/N) of the parents, parental satisfaction with comprehensive care (VAS)
- *Children*: age, type of SMA (multiple choice), age at onset of first symptoms, age at diagnosis, type of therapy, time between treatment start and research participation (calculated variable),
- *Therapeutic decision*: parental participation in therapeutic decisions (Y/N) and satisfaction that their opinion has been taken into account by the medical team (VAS)
- *Treatment effects*: effects observed by the parents following treatment (Y/N), effects on motor function (multiple choice) and autonomy (Y/N), satisfaction with the observed changes (VAS), evolution of parental concerns regarding the health and future of the child (multiple choice) and evolution of the feeling of parental overload related to the disease and its management (multiple choice)

(2) Variables from the self-questionnaires evaluating the medium-term psychological consequences on parents: depression—Beck Depression Inventory, BDI-II (15), state-trait anxiety—State-Trait Anxiety Inventory, STAI-Y (16, 17), parental stress—Parental stress Index short form, PSI SF (18), sense of coherence—Self-questionnaire, SOC-13 (19), parental burnout—Parental burnout questionnaire, PBA (20), marital satisfaction—Dyadic Adjustment Scale, DAS 16 (21), impact of the child's disability on the family—Family impact of Childhood Disease, FICD (22, 23).

(3) Variables from semi-structured interviews transcribed and analyzed with N'Vivo: “Therapeutic decision-making” sub-node.

### Data analysis

Quantitative data from the in-house self-questionnaire and self-assessment scales were analyzed using SAS software (version 9.4; SAS Institute Inc., Cary, NC, USA). Data were reported as counts and frequencies (%) for qualitative variables and mean ± standard deviation for quantitative variables.

The thematic analysis allowed us to identify two parental attitudes towards decision-making: “hesitant parent” and “non-hesitant parent”. We also identified a difference in parental ideas about gene therapy (GT) vs. other innovative therapies. This allowed us to perform a descriptive statistical analysis by subgroup using “hesitant” parents (*n* = 7) vs. “non-hesitant” (*n* = 11) parents and parents whose child received GT “GT-treated” (*n* = 6) vs. those with a child treated with “other therapy” (*n* = 12). The homogeneity between the subgroups was tested using the appropriate tests: the *χ*^2^ test (or an exact Fisher test) for qualitative variables, and a Student test (or non-parametric Mann-Whitney test according to the distribution variable) for quantitative variables. The results were considered statistically significant when *p* ≤ 0.05.

The interviews were transcribed and imported into qualitative data analysis software NVivo 10 (version 1.6.1). The encoding map was produced jointly by three experienced psychologists using deductive methodology: two main thematic nodes were defined based on the literature and the objectives of the SMAPAR study: “Parenting” and “Parental overload”.

We then moved on to an inductive phase: three psychologists (SB, MA, and MG) analyzed the first three interviews together in order to identify the emerging themes, define the sub-nodes to organize the data hierarchically, and check internal validity. A research psychologist (SB) then coded the other 15 interviews from the coding map, which was gradually improved by the iterative interpretative approach. Rereading the interviews allowed us to verify and specify the sub-nodes. We present an analysis of the “therapeutic decision-making” sub-node, created within the parent node here: Parenting → Discovery of SMA → Therapeutic decision-making and it's 5 themes, some of which are broken down into sub-themes.

The interview guide and research methodology were pilot tested on one mother and one father prior to the study and adjusted according to their feedback. Excerpts of the interviews were translated to English to be used in this paper.

## Results

### Participants

The sample (Table 1) consists of parents of a child with SMA1 treated with an innovative therapy (*n* = 18), including 12 mothers (66.7%) and 6 fathers (33.3%). The average age of the parents was 34.7 years (±5.2); 15/18 parents (83.3%) were married; 3/18 parents (16.7%) were separated from the other parent of the affected child at the moment of assessment (2/3 had another partner and 1/3 were single); 10/18 parents (55.6%) participated in the research jointly as a couple, 10/18 parents (55.6%) were first-time parents. The mean age of the child as reported by the parents was 44.3 months (±38.0); 10/18 parents (55.6%) reported that their child was classified at type 1 SMA and 8/18 (44.4%) of type 1c. They evaluated the average age of onset of symptoms at 3.5 months (±2.3) and diagnosis at 5.4 months (±3.2). The average time between the introduction of innovative therapy and research participation was 813.6 days (±455.2). The different therapies and management options proposed to the 18 parents are divided as follows: 11 parents (61.1%) innovative therapy at the time of diagnosis, 2 (11.1%) participation in a clinical trial, 2 (11.1%) a choice between a clinical trial or an innovative therapy, 2 (11.1%) a palliative (end of life) care, and 1 parent (5.5%, child 1c) was offered pro-active symptomatic care, as therapies were not yet available at the time of diagnosis. Finally, all children of participating parents had either received or were receiving innovative therapy at the time of the study. Note that 2/18 (11.1%) parents reported that their child was not treated, although the two children received a gene therapy injection. Regarding to the parents' socio-economic status, 2/18 parents (11.1%) were considered “low-income”, 9/18 (50.0%) “working class”, 5/18 (27.8%) “middle class” and 2/18 (11.1%) “high-income”. Among the parents, 13/18 (72.2%) were working and 5/18 (27.8%) were unemployed; 15/18 parents (83.3%) received social assistance related to the child's education and or child's disability.

**Table 1:** Characteristics of participants (*n* = 18, homemade self-reported questionnaire).

All parents	Total *n* = 18	Non-hesitant *n* = 11	Hesitant *n* = 7	GT-treated *n* = 6	Other therapy *n* = 12
Gender of parent					
Male	6 (33.3%)	3 (27.3%)	3 (42.9%)	1 (16.7%)	5 (41.7%)
Female	12 (66.7%)	8 (72.7%)	4 (57.1%)	5 (83.3%)	7 (58.3%)
Age (years)	34.7 (±5.2)	32.6 (±5.1)	38.0 (±3.3)	32.8 (±6.8)	35.7 (±4.1)
Marital status					
Married	15 (83.3%)	8 (72.7%)	7 (100%)	6 (100%)	9 (75.0%)
Separated and in couple	2 (11.1%)	2 (18.2%)	-	-	2 (16.7%)
Separated and single	1 (5.6%)	1 (9.1%)	-	-	1 (8.3%)
Participation in couple					
No	8 (44.4%)	5 (45.5%)	3 (42.9%)	4 (66.7%)	4 (33.3%)
Yes	10 (55.6%)	6 (54.5%)	4 (57.1%)	2 (33.3%)	8 (66.7%)
Primiparous					
No	8 (44.4%)	2 (18.2%)	6 (85.7%)	1 (16.7%)	7 (53.8%)
Yes	10 (55.6%)	9 (81.8%)	1 (14.3%)	5 (83.3%)	5 (41.7%)
Economic status					
Poor	2 (11.1%)	2 (18.2%)	-	1 (16.7%)	1 (8.3%)
Popular	9 (50.0%)	6 (54.5%)	3 (42.9%)	4 (66.7%)	5 (41.7%)
Medium	5 (27.8%)	3 (27.3%)	2 (28.6%)	1 (16.7%)	4 (33.3%)
Easy	2 (11.1%)	-	2 (28.6%)	-	2 (16.7%)
Parent's working					
No	5 (27.8%)	2 (18.2%)	3 (42.9%)	2 (33.3%)	3 (25.0%)
Yes	13 (72.2%)	9 (81.8%)	4 (57.1%)	4 (66.7%)	9 (75.0%)
Social benefits					
No	3 (16.7%)	1 (9.1%)	2 (28.6%)	1 (16.7%)	2 (16.7%)
Yes	15 (83.3%)	10 (90.9%)	5 (71.4%)	5 (83.3%)	10 (83.3%)
Age of child (months)	44.3 (±38.0)	46.5 (±48.0)	40.7 (±15.2)	25.2 (±3.4)	53.8 (±44.0)
Type of SMA					
Type 1	10 (55.6%)	7 (63.6%)	3 (42.9%)	3 (50.0%)	7 (58.3%)
Type 1 c	8 (44.4%)	4 (36.4%)	4 (57.1%)	3 (50.0%)	5 (41.7%)
Age of symptoms’ onset (months)	3.5 (±2.3)	3.5 (±2.7)	3.6 (±1.8)	2.8 (±1.5)	3.9 (±2.7)
Age of diagnostic (months)	5.4 (±3.2)	5.5 (±3.7)	5.3 (±2.4)	5.3 (±2.5)	5.5 (±3.6)
Treated with innovative therapy					
Answer no, but it was yes	2 (11.1%)	2 (18.2)	-	2 (33.3%)	-
Yes	16 (88.9%)	9 (81.8%)	7 (100%)	4 (66.7%)	12 (100%)
Type of innovative therapy					
Gene therapy	6 (33.3%)	5 (45.5%)	1 (14.3%)	-	-
Other therapy	12 (66.7%)	6 (54.5%)	6 (85.7%)	-	-
Time from research participation to start of treatment (days)	813.6 (±455.2)	666.8 (±375.9)	1002.3 (±505.7)	586.0 (±118.6)	889.4 (±503.6)

### Data from the in-house self-questionnaire

The results of the descriptive analysis are presented in Table 2. When asked about their participation in therapeutic decision-making (treatment and care) for their child, only 1/18 parents (5.6%) did not feel involved. The parents felt their opinion was considered in the medical teams' decision with an average score of 80.6/100 (±16.6). All parents who reported that their child had received innovative therapy (*n* = 16) observed changes upon introduction of the treatment, including improvement in motor function (16/16, 100%), acquisition of new motor functions (12/16, 75.0%) and increased autonomy (10/16, 62.5%). Parental satisfaction with the observed improvements is on average 71.7/100 (±17.7). Despite the observed improvements, disease burden decreased for only 3/16 parents (18.8%), remained stable for 10/16 parents (62.5%) and increased for 3/16 parents (18.8%). Concerns related to the child's health decreased for 6/16 parents (37.5%), remained stable for 8/16 parents (50%) and increased for 2/16 parents (12.5%). Concerns for the child's future increased for 5/16 parents (31.3%), remained stable for 8/16 parents (50%) and decreased for 3/16 parents (18.8%). The average score of parents, when asked about their overall health satisfaction, was 67.3/100 (±20.8). However, 16/18 of parents (88.9%) believe that SMA has impacted their physical health and 12/18 (66.7%) their mental health. There is no significant difference between the subgroups of “hesitant” parents vs. “non-hesitant” parents and the subgroups of “GT-treated” vs. “other therapy” for all the variables mentioned above.

**Table 2:** Quantitative data from the in-house self-questionnaire (results are expressed as mean score ± standard deviation).

All parents	Total *n* = 18	Non-hesitant *n* = 11	Hesitant *n* = 7	*p*-value	GT-treated *n* = 6	Other therapy *n* = 12	*p*-value
Involvement in medical and therapeutic decision process				0.412			0.146
No	1 (5.6%)	1 (9.1%)	-		1 (16.7%)	-	
Yes	17 (94.4%)	10 (90.9%)	7 (100%)		5 (83.3%)	12 (100%)	
Satisfaction with the way their views were taken into account	80.6 (±16.6)	77.7 (±9.8)	84.7 (±23.7)	0.137	77.6 (±22.8)	81.8 (±14.4)	1.000
Satisfaction about their overall health	67.3 (±20.8)	63.1 (±14.6)	74.0 (±28.0)	0.291	62.0 (±20.5)	70.0 (±21.3)	0.458
Parents whose physical health is affected by the SMA				0.060			0.289
No	2 (11.1%)	-	2 (28.6%)		-	2 (16.7%)	
Yes	16 (88.9%)	11 (100%)	5 (71.4%)		6 (100%)	10 (83.3%)	
Parents whose psychological health is affected by the SMA				0.087			0.289
No	6 (33.3%)	2 (18.2%)	4 (57.1%)		1 (16.7%)	5 (41.7%)	
Yes	12 (66.7%)	9 (81.8%)	3 (42.9%)		5 (83.3%)	7 (58.3%)	
**Parents who declared their child was treated**	***n* = 16**	***n* = 9**	***n* = 7**	** **	***n* = 4**	***n* = 12**	
Observed change related to treatment: Yes	16 (100%)	9 (100%)	7 (100%)		4 (100%)	12 (100%)	
Type of motor changes observed				0.771			0.182
Motor function improvments	16 (100%)	9 (100%)	7 (100%)		4 (100%)	12 (100%)	
And/or New motor function acquisition	12 (75.0%)	7 (77.8%)	5 (71.4%)		4 (100%)	8 (66.7%)	
Changes in level of concern about their child's health				0.881			0.641
Increased	2 (12.5%)	1 (11.1%)	1 (14.3%)		-	2 (16.7%)	
Same	8 (50.0%)	5 (55.6%)	3 (42.9%)		2 (50.0%)	6 (50.0%)	
Decreased	6 (37.5%)	3 (33.3%)	3 (42.9%)		2 (50.0%)	4 (33.3%)	
Changes in level of concern about their child's futur				0.866			0.915
Increased	5 (31.3%)	3 (33.3%)	2 (28.6%)		1 (25.0%)	4 (33.3%)	
Same	8 (50.0%)	4 (44.4%)	4 (57.1%)		2 (50.0%)	6 (50.0%)	
Decreased	3 (18.8%)	2 (22.2%)	1 (14.3%)		1 (25.0%)	2 (16.7%)	
Satisfaction with the changes observed following treatment	71.7 (±17.7)	70.3 (±14.8)	73.4 (22.1)	0.741	74.3 (±18.3)	70.8 (±18.3)	0.751
Changes in burden due to the SMA and its management				0.660			0.537
Increased	3 (18.8%)	2 (22.2%)	1 (14.3%)		1 (25.0%)	2 (16.7%)	
Same	10 (62.5%)	6 (66.7%)	4 (57.1%)		3 (75.0%)	7 (58.3%)	
Decreased	3 (18.8%)	1 (11.1%)	2 (28.6%)		-	3 (25.0%)	

### Evaluation of medium-term psychological effects on parents' lives

The results of the descriptive analysis are presented in Table 3. Overall, the parents do not have high depression scores (mean BDI score: 9.8 ± 6.1), 5/18 parents (27.8%) presented with mild depression and 1/18 parent (5.5%) with moderate depression. The sub-group analysis highlights that: the “non-hesitant” group of parents presented mild depression (13.2 ± 4.3) as compared with the “hesitant” group of parents who presented no depression (4.4 ± 4.5) (*p* < 0.001); the group of parents of a “GT-treated” child also presented mild depression (15.0 ± 3.6) as compared with the group of parents of child treated with “other therapy”, which presented no depression (7.2 ± 5.4) (*p* = 0.013).

**Table 3:** Medium-term psychological effects on parents’ lives (results are expressed as mean score ± standard deviation).

All parents	Total *n* = 18	Non-hesitant *n* = 11	Hesitant *n* = 7	*p*-value	GT-treated *n* = 6	Other therapy *n* = 12	*p*-value
Depression (BDI-II)	9.8 (±6.1)	13.2 (±4.3)	4.4 (±4.5)	<0.001	15.0 (±3.6)	7.2 (±5.4)	0.013
State anxiety (Stai-YA)	39.3 (±8.8)	43.0 (±7.6)	33.6 (±7.9)	0.022	42.7 (±4.2)	37.7 (±10.2)	0.270
Trait anxiety (Stai-YB)	40.7 (±9.7)	45.1 (±7.1)	33.7 (±9.5)	0.010	45.5 (±4.4)	38.3 (±10.8)	0.139
Parental stress (PSI-SF)	82.9 (±22.0)	91.8 (±17.9)	69.0 (±21.7)	0.027	83.7 (±18.1)	82.6 (±24.5)	0.925
Parental burnout (PBA)	26.7 (±19.0)	30.0 (±18.9)	21.6 (±19.4)	0.374	27.0 (±25.1)	26.6 (±16.4)	0.967
Sense of coherence (SOC-13)	57.8 (±12.5)	53.8 (±10.7)	64.1 (±13.4)	0.088	56.8 (±8.5)	58.3 (±14.5)	0.819
Dyadic adjustment (DAS-16)	114.4 (±21.8)	103.3 (±18.7)	127.2 (±18.5)	0.076	110.2 (±4.2)	117.2 (±28.2)	0.486
Negative impact of the disease on family life (FICD −)	27.4 (±6.3)	29.2 (±6.2)	24.7 (±5.9)	0.290	27.7 (±6.7)	27.3 (±6.4)	0.920
Positive impact of the disease on family life (FICD +)	33.2 (±4.8)	32.5 (±5.1)	34.3 (±4.3)	0.475	33.3 (±4.3)	33.2 (±5.2)	0.947

With regard to anxiety, the parents presented a low level of trait (YB) and state (YA) anxiety on average (mean score Stai YB = 40.7 ± 9.7 and YA = 39.3 ± 8.8); 6/18 parents (33.3%) and 7/18 parents (38.9%) presented average trait and state anxiety scores respectively.

The sub-group analysis highlights that: the group of “non-hesitant” parents presents mild anxiety (average score YB = 45.1 ± 7.1; YA = 43.0 ± 7.6) compared to the group of “hesitant” parents who presents very low anxiety (mean score YB = 33.7 ± 9.5; YA = 33.6 ± 7.9) (*p* < 0.05); the parents in the “GT-treated” group show mild anxiety (mean score YB = 45.5 ± 4.4; YA = 42.7 ± 4.2) as compared with parents in the “other therapy” group who had very low anxiety (mean score YB = 38.3 ± 10.8; YA = 37.7 ± 10.2). There is no significant difference between the 2 groups.

At the time of assessment, the parents did not show significant parental stress (average PSI-SF score = 82.9 ± 22.0). However, according to establish criteria of the scale, 11/18 parents (61.1%) did suffer from clinically significant levels of stress. The analysis by subgroup underscores that the “non-hesitant” parents presents a clinically significant level of parental stress (average PSI-SF score = 91.8 ± 17.9) unlike the “hesitant” group who does not present parental stress (score mean PSI-SF = 69.0 ± 21.7) (*p* = 0.027). The parents in the “GT-treated” group and the parents in the “other therapy” group did not show significant parental stress (mean PSI-SF score = 83.7 ± 18.1 and 82.6 ± 24.5, respectively). There is no significant difference between the two subgroups.

As a group, the parents presented no particular risk of parental burnout (average PBA score = 26.7 ± 19.0). However, taken individually, 2/18 parents (11.1%) presented a low risk of parental burnout and 5 parents (27.8%) a moderate risk. The mean internal consistency score (SOC-13 score) was 57.8 ± 12.5 and overall, the parents showed a good level of dyadic adjustment within their relationship (mean DAS 16 score = 114.4 ± 21.8). The subgroup analysis for these 3 scales indicates that there are no significant differences between the different subgroups.

Finally, examination of the impact of disability on family life (FICD) reveals both a moderately negative impact (27.4 ± 6.3) and a strong positive impact (33.2 ± 4.8). There was no significant difference between the different subgroups.

### Qualitative data from semi-structured interviews

Thematic analysis of the interviews allowed us to define 5 themes within the “Therapeutic decision-making” node, some of which were further broken down into sub-themes.

#### Theme 1: Experience of the treatment proposal

##### The doctor's ability to deliver complete and reasoned information

According to the parents, how the neuropediatrician proposed the therapeutic strategy seemed to be based on his experience with clinical trials and new therapies. The parents found that doctors made proposals in a measured way. Their manner of proposing treatment on the one hand hinted at the epistemic uncertainty surrounding the innovative therapies, while also conveying confidence in their acquired experience since the beginning of use of the new therapies: both in terms of effectiveness as well as with regard for the clinical state of the child.

*“The therapeutic trial was explained to us fairly well; we knew very well that there was no miracle. It was clearly explained to us that it would not cure the disease, but that it would reduce its effects. That being said, we had no idea what we were getting in to and we also knew that there was a part of the trial that used placebo”* (21)

*“He explained to us that there was a new drug that had arrived which had been through a phase of tests and that with this drug, maybe he would live beyond two years, even up to 50 years, we didn't know. But he explained that there were not enough long-term results”* (17)

*“We talked about it a bit with the doctor. When he told us about the appointment that had been made for the gene therapy trial, we said to him “please give us your opinion, we'll take it!” He was quite courageous, because he said that if it was for his children, he would do it without hesitation, he would not ask questions. So, it reassured us from a medical point of view, to say to ourselves “We don't know anything about this, but if we look at this person who is very interested and informed, and seems very professional, he would do it!”* (10)

*“According to him, our son was not particularly ill, and it also did not seem to be a question of him not receiving treatment […] Anyway, I think that if we had absolutely wanted him not to be treated, I don't think the doctor would have necessarily objected. But to him, our son was a really good candidate for innovative therapy”* (13).

##### Doctors who convey incomplete information or false hopes

Conversely, some parents reported that when treatments were discussed at the time of diagnosis by doctors who they later perceived as less informed, the information was sometimes incomplete, and conveyed a prediction of the future or unreasonable hope.

*“They were unable to tell us what we could expect with the therapy. At that time, she was already nine months old, so we said to ourselves: “we have three months left””* (11)

*“The doctor told me about gene therapy and said, “we will be able to cure your son”. And I asked him “what do you mean cure him? She replied: “Ma'am, he will be able to walk! “. That's the only thing I retained. But when we went to meet another doctor, at the expert center, it was a rude awakening. She examined him and told us that she did not think he was particularly affected and that she wasn't sure if the therapy would work on him, she was very honest and she gave us a few days to consider our options”* (41)

##### The sense of urgency in decision-making

Given that the prognosis of the child was at stake, the parents' testimonies highlight the context of emergency in which the decision was made. The parents recounted that the doctor conveyed the need to treat the child as quickly as possible just after making the diagnosis.

*“She told us that she was giving us a little over 24 h to think things over, because we had to act quickly. At that moment, my husband and I looked at each other and we said to ourselves: “It's not worth it, we are not going to wait 24 h. If there is something to try, we'll do it now…”* (41)

*“The doctor said: “But we really have to make up our minds because it's a race against time and you have to treat your son now, he can't wait any longer, we don't have time.”* (13)

##### Parental ideas about innovative therapies

At the time when the decision was made, the parents remember having had vague ideas about innovative therapies. Some parents understood that these treatments would not cure their child and that there was uncertainty about the expected effects. Others evoke the idea of a miracle or a cure that had been conveyed by certain professionals, social networks, and/or the media. In addition, the parents who were offered gene therapy mentioned greater efficacy and a less restrictive mode of administration than the other alternatives. They also cite a potential risk of greater side effects, but this risk does not tip the balance on their final decision.

*“We knew that it was partly random, we didn't know why it worked better for some children than for others”* (13)

*“The doctors told us: “She will be able to walk”. So, they ignited hopes that were unrealistic at the time”* (60)

* “I asked him, “What's the difference between Spinraza and Gene therapy?” Is there really a noticeable difference? “She said to me: “When it is introduced before symptoms, there is a real difference” And I also said to myself: “He won't have to have epidurals every four months in his spine”, I found it less burdensome on a daily basis for our family, for our child.”* (44)

*“At the time, I think we said to ourselves, it's a bit like a magic wand. And they're going to give us back our little girl, the one we had when she was born.”* (32)

*“We had some fears: won't this have other effects that we have no idea about today? Because it goes in the blood, we do not know exactly what other risks might be associated with the treatment”* (10)

#### Theme 2: Parental attitudes at the time of the decision

##### Accepting without hesitation to save the child's life at all costs

Among the 16 parents who received approval for an innovative therapy, 9 parents accepted that their child be treated unequivocally regardless of the proposed strategy.

*“Spinraza had been on the market for about six months. So here it is! We didn't hesitate, we were told about it and we said: “Well, if there's already a treatment, we're not going to take the risk of doing nothing.”* (14)

*“Since it was that or nothing, well we went for it”* (21)

*“We had understood that without innovative therapy, he would not make it to age 2 and that, maybe with this treatment, he would get better. He might never be able to walk, but maybe he could live. So, if there was something to try, it was logical that we would try it.”* (41)

*“We said to ourselves, we are going to put all the odds in his favor. In any case, we had nothing to lose. We were told he was going to die before the age of 2”* (17)

##### Hesitating before making a decision

Some parents (*n* = 7) hesitated between pursuing a treatment or pursuing end of life care due to the uncertainty, the seriousness of the disease, and the fear of imposing a life of suffering on their child in the form of severe disability.

Several mention the risks of both options: (i) pursuing palliative care might also mean taking the (low) risk that the child will survive with a severe disability; (ii) accepting innovative therapy means accepting the risk that the treatment will not be effective, particularly if symptoms were already present. The parents highlight the dilemma they had to face between two solutions marked both by uncertainty.

Among the 7 hesitant parents, 2 parents had initially chosen to refuse the treatment proposal, then changed their minds after a second opinion from an expert center where they had been informed that their child suffered of a later and more slowly progressive form of SMA (1c) and that he would most certainly survive for years to come, even without innovative therapy.

*“I said to myself: “Before doing anything, we will have to have very specific opinions on: What state is he currently in? What can we expect and what should we do? For my part, I wasn't ready to let him live at any cost””* (44)

*“My husband and I didn't really know what to do because at the time, it was not presented as a treatment. It was more palliative, more as support for a death without suffering, in fact. So, my husband and I took 48 h to respond, we didn't really know what to do.”* (17)

*“We still ask ourselves the question, “is it this the life that I want or not? “We were still given the possibility of doing nothing and accompanying him towards his end…. with a small risk that he will in fact live, with a huge handicap and without being able to do anything. We ask ourselves all of these questions because having a disability is not the life we had wanted for us, nor for him”* (9)

“*In the beginning we were told that this little girl, she was going to live less than a year that she was surely going to have a tracheostomy, a gastrostomy that her heart was in danger of stopping. And we said to ourselves: “Okay, but we aren't going to impose additional treatment on her” […]. We said to ourselves “this is still a very important question for us, so we need a second opinion.” And in fact, when we saw the doctor, he told us: “Of course she needs to be treated [….] in my opinion, without treatment she can live until the age of three”. And we said to ourselves “there are actually children like her, adults like her and maybe, even with this disease, she will be able to have her own child. In the end, we suddenly fell in love with the idea of her destiny and that really made the decision for us”* (11)

#### Theme 3: Whose decision is it?

##### A shared decision between parents and doctors

The majority of parents feel that they took part in the decision thanks to a trusting relationship with the medical teams.

*“In general, I think that the, well, I think we can talk a little about collaboration between the medical team and ourselves because, yeah, we are involved, and they are involved as well. She is really very, very good, we are really…Well, we realize how lucky we are to have this doctor and this whole medical team”* (13).

##### Parental responsibility for the decision

For 5 parents, the decision was a radical choice between the life and death of their child. Faced with such a dilemma, parents reacted in a variety of ways: 1 couple chose to delegate their decision to the doctor, as they found it to be an impossible responsibility, another couple shifted the decision-making to their child, somehow entrusting him with the responsibility to choose whether he wanted to live or not, and finally, 1 parent took on the responsibility to make the decision with his partner, but not without guilt.

*“It did not seem to us to be our role as parents to have to make a choice, to say he must have this therapy, or he must have this other therapy. So, for us it was clear. And for the doctor it was also clear that ultimately he would be the one to make the decision”* (12)

*“We agreed that we didn't want to choose between life and death because it's not up to us to choose, it would be up to him. And despite his young age, I think children if they want to live, they live, if they don't want to live, they don't live. We said to ourselves, we're going to put the odds on his side.”* (17)

*“There are very few cases in life where you are responsible for the life or death of your child. We, however, gave birth to a child with a fatal illness. So already, there was an extra weight on us. And afterwards, we said to ourselves: “Are we forcing him to live? And at what cost? “but it's still us who have to decide. So twice in a row, we said to him: “We gave you this life, we will make you survive”* (44)

##### A feeling of dispossession, an imposed decision

In our study, 3 parents felt that the decision had been imposed upon them either because the choice of therapy was made by the MCM without taking into account the parents' viewpoint, or because they had not received approval for their child to receive an innovative therapy.

When palliative care is proposed but the parents prefer their child to be treated, it feels like a door slammed shut. These parents believe that the decision was imposed upon them, and they experience it as a theft of their parental authority.

*“She explained to us that in any case, it is not us who decide on the therapy she will be able to have. It is a group of doctors who decide after analyzing […] We understood that it was really the doctor who decides because it was ultimately a medical decision”* (32)

*“Clearly, we were advised against it, we were not told: “You can try”, we were told: “No. Don't do it” right?”* (3)

*“Uh, he didn't advise us at all. In actual fact, we were not given a choice. We were told, “Gene therapy, don't count on it. And Spinraza, we submitted an application and in committee, it wasn't accepted because he was too ill”* (4)

#### Theme 4: Seeking information and support from the medical teams

##### The internet and patient associations

After the diagnosis and treatment proposal, one of the main sources parents report finding information is the Internet. They report feeling a need to learn about the disease and new therapies on French and international websites. They also hope to find other more detailed information or testimonials from other parents. Several parents consulted patient association sites or were referred to them by their doctor to find out about the disease, or to seek support when palliative care was being considered. In this specific case, contact with a patient association allowed a parent to develop his ideas about palliative care thanks to a conversation with a parent who had been through it with his child. It also allowed some parents who had initially been offered palliative care to seek a second opinion at an expert center.

*“We looked for a lot of information on the internet: what is this treatment? What is gene therapy? There was a study in the United States two or three years ago, on 12 children. So, we were looking for all the documents which we were then trying to translate. Our life revolved entirely around that”* (9)

*“I had a conversation on the phone with someone who told me about the death of his child… so I asked him a lot of questions about the circumstances of his death, and it did me a lot of good. It was interesting because we needed to hear from other parents who had been through a similar experience”* (11)

*“We said to ourselves, we are going to meet parents who this has happened to, we'll discuss their experience in order to have some moral support for being with our child in end-of-life care. It was my wife who contacted the association, the woman asked her questions about our son's health and right away, she suggested a second opinion. On my end, I didn't necessarily believe it was worth it and that's when my wife was put in touch with another mother who put us in touch with her neuropediatrician”* (9)

##### Support from the medical teams

Overall, the majority of parents who were offered innovative therapy said they felt supported by the medical teams throughout the decision-making process. They describe professionals with a sense of humanity who were available and committed to them. Nevertheless, one parent highlighted the difficulty of interpreting the quantity of information given to them at the time of diagnosis. In contrast, the couple who was initially offered palliative care evoked the lack of support from the medical team when they challenged their decision and obtained treatment in another center.

*“When we made our decision, it was also about the support from the team. We knew we would have the answers to our questions that we were really given all the information we needed. But it was up to us to interpret and understand the information how we wanted to understand it… I'm not saying that the support was the best”* (17)

*“They never had the same perspective as we did. We were in action mode while they were in end-of-life mode. And when our son was not doing well, even then, they, they would say, “well, we told you so. You shouldn't have expected anything else! Since his pediatrician didn't believe in the treatment, well suddenly everything she said was pushing in that direction.”* (3)

#### Theme 5: The experience of decision-making in the medium term

##### Satisfaction with the observed effects

The majority of parents describe themselves as satisfied with the progress they have observed in their child following treatment. This satisfaction is essentially linked to their child's motor progress and improvement in respiratory function. Note that their satisfaction is not related to the child's autonomy. The fact that the child was able to survive is deemed a miracle by some parents and a source of hope despite the child's severe disability. On the other hand, five parents describe mixed satisfaction with the treatment: because the child encountered medical problems following its implementation, because the child's progress is not up to the standard of parental expectations, or because signs of the disease appeared unexpectedly to the parents.

*“It's been three weeks since we started a new treatment and we still have the impression that he talks a bit, well he babbles a lot more than before. He's starting to be able to turn from his back on both sides and come back on his own. He's been in really great shape since December and that's been really good for us.”* (13)

*“Right now I have a four-year-old girl, not one with spinal muscular atrophy, but a four-year-old girl with a motor delay”* (14)

“*She is 5 and a half years old, it's a miracle. It's awesome. It is indescribable. Finally, I'm actually happy”* (22)

* “So, he can't hold his head up, he can't stay seated or anything at all really, but hey, well, we can still see that the treatment is working and that something is happening. So, we're being patient and we're telling ourselves that over time it will happen”* (3)

*“He can sit up, but we can't leave him sitting alone, because we're still afraid that he will fall. So, it's more of a type 1, but we still have the life of parents who have a young type 2. So, we say to ourselves: “At the moment, it is how it is, even if it doesn't evolve positively, it won't evolve negatively.”* (44)

*“There are things that are going well and there are things that can't be controlled. And scoliosis is one of the things that's described, especially when the treatment comes a little later on”* (10)

##### A decision that doesn't go away

In retrospect, parents perceive the diagnosis as confronting them with the helplessness and uncertainty of a future marked for premature death or severe disability, their discourse evokes decision-making as the only way of taking action. That being said, parents report that when making the decision, they did not understand all of the issues relating to their child's illness and its treatment.

At the time the decision had to be made, it was impossible for the parents to imagine what the future of their child might be or the impact of the disease on their daily and family life in the short and medium term. It was also impossible for them to envisage how end of life care for their child would be.

According to the parents, it is not as if the decision is final, but rather a decision that continues to be relevant and which they will reexamine at different times throughout the course of care or the development of the child. This is particularly true when the child's health is worrying and the risk that he or she suffers from a severe disability is confirmed.

In these moments, the parents wonder if the decision to support their child in palliative care would not have been better and 4 parents mention their fear that their child will 1 day blame them for the choice that they have made on their behalf.

*“Very quickly we were told: “You have the opportunity to do something!”. And then the first day, it's like a huge void. But we reacted fast because we knew that we had to act quickly in order to succeed in getting the most decent life possible for him, so we moved to action quite quickly.”*(9)

*“At the time I had not realized the impact on respiratory function. When I was told muscle, I didn't think of that. And swallowing was ultimately much more significant than the impact on motor muscles. So, it's true that at the time, I didn't understand what it was going to produce in terms of breathing and swallowing and that's our son's main issue.”* (13)

*“I told them, “Listen! I can survive the death of my child”. But then, witnessing the end of his life, I really don't know how it will happen, I don't know if I will be able to do it. And I told them, “Listen! If it doesn't go well, I'll smother him under a pillow”* (44)

*“I hadn't understood what it would entail: this amount of care, of time throughout the day…. or anything really. I didn't realize, what it was going to mean in my life, you know?”* (22)

*“It's true that seeing your child suffer, being unwell, to have the impression that it's something chronic and that it's improving, and then they relapse. At certain times we ask ourselves: “were all these efforts really necessary and is treatment necessarily the best solution? “Then again, we also don't have the impression that it is relentless or overly aggressive”* (12)

*“It happens less and less, but there are still times when I say to myself: “Oh my God! I would rather he was dead, you know?” Well, even now […] I think it'll only be when he's an adult and he can say to me: “Wow, Mom! It was so worth it” that I will could think to myself, “We made the right decision.”* (44)

## Discussion

This is the first study in France to examine how parents of children with SMA type 1 experience the decision-making process around innovative therapy for their child. The participants in our study were mainly mothers, which is consistent with participation rates found in the literature (24). The average age of parents in our study is slightly higher than the average age reported in the only other study providing information on the age of parents of children with type 1 SMA (25). Most parents in our sample were married and reported a good level of marital satisfaction. We believe that the young age of their children and the hope that a treatment offer provides may have a protective effect on the couple, an effect which remains to be demonstrated over the long term.

Parents describe a high degree of satisfaction with the effects observed in their child following the start of treatment as is consistent with caregivers' evaluations found in the literature. According to Audic et al., parents seem to be overly optimistic; this over-optimism may reflect the considerable hopes invested in these new therapies (8). Despite this, when asked if the burden related to the disease and the care of their child had decreased since starting treatment, results show that it only decreases for a minority of parents. In our study, we did not measure parental responsibility quantitatively as we felt there were no relevant tools to do so. Brand et al. point out that studies based on quantitative methodologies report an average to moderate burden (24, 26–29) in parents of children with SMA. However, these studies used the Zarit scale or the Caregiver Strain index, scales initially developed to assess the burden of caregivers of patients with dementia and/or the elderly and which have not been validated in a population of parents of children with degenerative diseases (30, 31). In addition, we believe that the many cares related to their child's disease carried out by the parents of our study might be perceived as expected for the care of any young child whose autonomy is limited, and therefore not perceived as a burden. In addition, a large proportion of the parents in our study were primiparous parents who could not have representations of what constitutes parenthood of a disease-free child.

The parents' satisfaction with the observed treatment effects also contrasts with their concerns with the future and health of their child. Concerns decreased for only 33.3% and 16.7% of parents after starting treatment. Indeed, despite the availability of innovative therapies, which change the course of the disease, a cure is still not possible (24). Uncertainty regarding the long-term efficacy of treatments raises ethical, medical, and financial questions for families (24). Accordingly, parental concerns are reported in the literature regarding the cost of innovative therapies, side effects and complications that therapies could induce (32, 33). These concerns also relate to the alteration in the quality of life of the child and their psychological distress linked to becoming aware of their difference and disability (32, 33).

The rate of depressed parents (33%) was found to be similar to that reported in the literature (34), but the level of depression in our cohort was mild to moderate, whereas Cremers et al. observed a high level of depression (34). On average, the low trait and state anxiety of our cohort contrasts with the high level of anxiety described in the literature among mothers (34). Note that the tool used by Cremers et al. to measure maternal depression and anxiety (HADS) is not the same as the one we used, limiting its comparability (34). Furthermore, the parents in the “hesitant” group are significantly less depressed (*p* < 0.001) and less anxious (*p* = 0.022) than those who were “not hesitant”, independently of their satisfaction with their child's progress after starting treatment. In our study, we attempted to determine the psychological states of the parents quantitatively using the BDI-2 and STAI scales. However, our qualitative approach leads us to believe that these scales cannot fully capture the very complex feelings parents report. Even though the interviews show similarities in themes, the experience reported by each parent was unique. The decision-making process seems to be very intimate and shaped by the personal history of each of the parents, which seems to be brought to the fore when confronted with such an extraordinary defy of parenting imposed on them by the disease. It seems important for us to point out that the majority of parents had never met with a psychologist and that this research was for many the first opportunity to recount their experiences at their own pace to someone with deep respect for their experiences. The psychologist who conducted the interviews (SB) perceived an intensely traumatic sentiment among the parents' accounts, particularly regarding the experience of misdiagnosis and then receiving a diagnosis, illustrating the intensity of what they have experienced on a psychological level. In addition, the psychologist herself experienced intense countertransferential feelings during the interviews, such as shock, blankness of thought, avoidance behaviours such as thinking about something else, and a feeling of helplessness. As the interviews progressed, she also described several somatic manifestations (fatigue, a feeling of physical overload), difficulties in getting back into the analysis of the interviews and a need to take care of herself. We believe that these manifestations are part of vicarious trauma, a term proposed by Laurie Pearlman and Karen Saatvine in the 1990s to describe the traumatic experiences of therapists working with survivors (35). It is now described in the therapeutical relationship, when the stories of patients have a traumatic effect on the therapist due to their repetition, which is associated with significant personal changes as a result of empathic engagement with the patient (35).

Furthermore, we might ask ourselves if the ambivalence at the time of the decision and the subsequent anticipation felt by some parents when presented with a treatment proposal may be protective. This could be pertinent for neuropediatricians who may perceive parental hesitation as an obstacle to decision-making. In reality, this first period of uncertainty could be considered necessary in order to integrate the complexity of their experience and begin the mental work needed throughout the evolution of the disease. Similarly, in the context of neonatology, it has been shown that parents who could anticipate difficulties during their pregnancy were better prepared to face them when the child had to stay in neonatology (36). It should also be noted that the parents of children who received gene therapy presented a significantly higher level of depression than those who received another therapy. This leads us to wonder if the proposal of gene therapy may induce an idealized vision of the treatment's effects or excessive hope of a cure, leading then to depressed mood when the parents are confronted with the current reality and forced to let go of that hope.

More than half of the parents reported high parental stress and more than a third of parents were at a low to moderate risk of developing parental burnout. The parental stress score is higher than that observed in the literature in parents of children and adolescents suffering from various neuromuscular diseases including SMA (37%, measured with the Parental Stress Scale) (37). According to Von Gontard et al. families of children with SMA, show high levels of stress compared to matched controls. The author points out that these levels of stress seem to be influenced by social support, the child's behavior, and the degree of disability (38). However, the use of different tools to measure parental stress makes the results difficult to compare and to our knowledge, no other studies have looked at parental burnout in parents of children with SMA. Furthermore, the “hesitant” group of parents did not present clinically significant levels of parental stress compared to the “non-hesitant” group. On the other hand, the parents of the “hesitant” group were not first-time parents. It is possible that having other healthy children may play a protective role in dealing with a new child's disease.

In our study, we adopted a salutogenic approach by focusing on the feeling of internal coherence: an important factor in psychological adjustment to stressful situations (39). The literature emphasizes that a high SOC score indicates better resistance to stress (39). The mean SOC score of parents in our study is lower than the mean score reported in a study that compared SOC in *n* = 368 parents of children with intellectual disability and/or autism and *n* = 387 parents of normal children (40). The literature points out that a weak sense of internal coherence is more strongly associated with the risk of parental overload, depression, and anxiety (39, 40), which does not seem to be the case in our research. However, the absence of norms to describe the SOC means we are not able to interpret its value in our population. Additional analysis to investigate the link between SOC scores and those of depression, anxiety, and parenting stress would allow us to see if the parents with the lowest SOC scores are also those with the highest depression, parental anxiety, and stress scores.

The fact that parents report a more positive than negative impact of the disease on family life, leads us to believe that the parents who agreed to participate in our study had a positive and combative attitude towards the disease. These results can therefore not be generalized to all parents, constituting a bias in our study.

The interview analysis reveals 3 determinants in the treatment decision-making process of parents of children with type 1. (i) **Giving your child every chance**: the literature on parents' expectations of the first approved therapy, Nusinersen, emphasizes that the majority of parents hoped that the treatment would modify the course of the disease, prolong the life of the child, improve their quality of life and allow the child to take charge of their illness and live with the most autonomy possible (24, 32, 33, 41); (ii) **Trust in the prescribing physician**. Kiefer et al. emphasize that the relationship with the medical team is the most important factor influencing parents’ final decision. The author specifies that to feel supported, parents need to receive sufficient information delivered in a neutral fashion with regard to the parent's decision by a doctor with expertise in the innovative therapies (33). Two parents in our study had initially chosen not to treat their child but changed their minds after seeking a second opinion from an expert center doctor who told them their child would not die. (iii) **Save the child's life and/or avoid being confronted with palliative care**, the notion of saving the child's life is never addressed in studies that have focused on therapeutic decision-making. In contrast, in their study on parental decision-making around the use of invasive ventilation in children with type 1 SMA in a palliative care setting, Pechman et al. point out that ventilation was the only possibility for parents to influence and prolong the life of their child (42, 43).

In our research it appears that retrospectively, parents believe they have been involved in medical decisions about their child. This finding is consistent with the work of Beenaert et al., which emphasizes that health professionals do not make decisions without informing parents (44). However, much like in our study in which several parents report having vague information, the author states that nearly 1/3 of the parents felt insufficiently informed about SMA and its treatments. Pechman et al. also point out that the parents of children with type 1 SMA diagnosed before new therapies, had not received sufficient information to understand all the issues surrounding the disease and palliative care at diagnosis. The authors emphasize that the traumatic context of the diagnosis and its repercussions are not conducive to understanding all the information. In addition, parents reported that doctors provided abstract and prosaic information that contrasted with the emotional intensity of their situation and led parents to seek further information from parent associations, social media, and the Internet (43). In contrast to these observations, a recent study conducted among parents who had received a neonatal diagnosis of SMA for their child reports that the advice given by the doctor had an impact on their decision for 83.3% of the participating parents and that it constituted the main influence over information from other health professionals, social networks, family and friends (45). In 2020, experts came to a consensus concerning the use of gene therapy in severe cases. They agreed that gene therapy could stabilize the disease without necessarily reducing disability or the child's quality of life (46). Following this publication, Gusset et al. were concerned that such a statement would leave little room for joint decision-making with families, would be misinterpreted by less experienced clinicians, would bias families' perspectives, and disrupt their decision-making process (47). The authors insisted on the need for the families concerned to be in contact with patient associations to develop realistic ideas about the life of a person and family with SMA and take part in medical decisions (47). In response to Gusset et al., Kirschner et al. highlight the need for parents not to be influenced in one way or the other. They emphasize the importance of explaining the wide range of outcomes associated with this therapy to parents, mainly because the public image is often idealized and seen as a cure, as in healing completely from SMA (46, 48). It should be noted that in our study, two parents stated that their child had not benefited from an innovative therapy when they had received gene therapy. When asked to elaborate on their response, the parents said that a treatment that did not cure the disease could not be considered as a treatment.

This surprising finding deserves more consideration with regard to families in long-term care as it is likely that the evolution of the disease will impose technical adaptations and a loss of function that the treatment will not be able to curb. Some parents may later feel disappointed and disillusioned with treatment and may consequently be reluctant to accept the prescribed medical recommendations to manage the child's disability. The innovative character of new therapies, in particular that of gene therapy, could be seen as a pharmakon (a philosophical idea denoting remedy, poison, and scapegoat). On the remedy side, it conveys hopes of healing, while on the poison side it induces fears about long-term side effects.

Retrospectively, several parents in our study reported that they had not understood all the issues around treatment when the decision was made and had constructed idealized representations based on what the doctor told them, information found on the Internet and/or transmitted by the associations, as well as their desire to save their child. We also noted that the families seem to have a perception of a hierarchy of innovative therapies leading them to prefer gene therapy as it seems to them more effective and less restrictive than other therapies despite the lack of long-term data on their safety and efficacy. Deng et al. (45) report similar findings about the criteria influencing therapeutic choice for parents of children who have undergone neonatal screening.

Our research has several limitations. First, like with most research on rare diseases, the size of our sample does not allow us to generalize these results to all parents of children with SMA type 1. Second, our study may be subject to selection bias, as the parent volunteers were mainly recruited from specialized consultations by family neuropediatricians and patient associations with close ties with the families.

## Conclusion

While the availability of innovative therapies is radically changing the management of SMA and the lives of families, the uncertainty of how well innovative therapies are tolerated over the long-term forces parents and medical teams to face particular dilemmas. In the absence of more reliable data and neonatal screening to initiate pre-symptomatic treatment, parents are faced with an impossible decision between the early death of their child and the risk that treatment will not allow their child to live independently.

The therapeutic decision appears to not remain in the past but instead reappears as the disease and its care progress. When observing their child's progress, the parents feel reassured. Conversely, when disability worsens or no progress is made, parents may wonder if they have made the right decision for their child.

The urgent context in which this decision takes place, just after the announcement of the diagnosis, highlights the importance of frank and open discussions with the doctor to provide comprehensive information and answer the questions that parents may have during the treatment proposal. Moreover, the urgent nature of this decision should spur medical teams to systematically propose a meeting with a psychologist with expertise in SMA whenever possible and at a minimum, after the moment of decision-making. In our opinion, this is an essential part of helping parents prepare for the sequence of decisions that will have to be made as treatment and the child's development progresses as well as for forging an alliance of trust between families and the medical team.

## Data Availability

The raw quantitative data supporting the conclusions of this article will be made available by the authors, without undue reservation. In compliance with the General Data Protection Regulation and to preserve the confidentiality of study participants, the raw qualitative data can't be shared.

## Footnotes

^1^Spinraza® (INN: Nusinersen) is an antisense oligonucleotide that increases the production of functional *SMN* protein by acting on the splicing of the *SMN2* gene. This is a very well tolerated treatment but requires an injection every 4 months into the cerebrospinal fluid via a lumbar puncture (intrathecal route) for life after a loading dose of 4 injections; Zolgensma® (INN: onasemnogene abeparvovec) is a gene therapy treatment designed to deliver the normal gene into defective motor neurons via a viral vector (AAV9). This is a one-time IV treatment that requires the injection of high doses of viral capsids that can cause serious side effects; Evrysdi® (INN: risdiplam) which is a motor neuron survival protein 2 pre-mRNA splicing modifier that increases the production of functional *SMN* protein. It is a very well tolerated oral treatment (syrup) requiring daily intake for life.

## Name of the SMAPAR Study Group

Karin Alvarez, Marco Araneda, Christine Barnerias, Audrey Benezit, Marie Bougeard, Nadia Blu Genestine, Odile Boesflug-Tanguy, Sophie Boursange, Ruxandra Cardas, Caudia Castiglioni, Jean-Baptiste Davion, Nayla Debs, Isabelle Desguerre, Silvana De Lucia, Elodie Deladrière, Emmanuelle Fleurence, Virginie Germa, Marta Gomez Garcia de La Banda, Marcela Gargiulo, Ariane Herson, Marie Hully, Arnaud Isapof, Javiera Jofré, Emmanuelle Lagrue, Laetitia Laïch, Julie Lejeune, Maripaz Martínez Jalilie, Marie-Christine Nougues, Laetitia Ouillade, Yann Péréon, Sarah Poncet, Claudia Ravelli, Susana Quijano-Roy, François Rivier, Marie Sarremejeanne, Caroline Stalens, Andrea Seferian, Bernadita Suarez, Caroline Wenzel and Denisa Zvarova.

## Author note

At the request of the publisher PAR we specify that : “*An unauthorized version of the French PSI/SF was used by the study team without permission. The PSI/SF is a copyrighted instrument and may not be used or reproduced in whole or in part, in any form or language, or by any means without written permission of PAR.*” We got a retroactive permission for administration of this version.
