# Supplementary material for: Parents' dilemma: A therapeutic decision for children with spinal muscular atrophy (SMA) type 1
Source: Front Pediatr. 2022 Dec 21;10:1062390. doi: 10.3389/fped.2022.1062390 (PMC9811815; doi:10.3389/fped.2022.1062390)
Supplement: Supplementary file 1 [file Datasheet1.pdf]

Supplementary Material

Methods of the SMAPAR Study

The SMAPAR study is an interdisciplinary collaboration with 4 follow-up consultations (3 in Paris and 1 in Chile) and Patients'association (AFM regional departments and ECLAS association).

The research was conducted independently and in parallel in France and Chile, using a common methodology. Each country is responsible for the analysis of its data and the comparison of the data between France and Chile will be done at the end of the research by the French team.

We conducted a mixed methods research, based on qualitative tools (in-depth semi-structured interviews) and quantitative tools (a self-administrated in-house questionnaire and 7 self-reported scales).

In France, 54 parents of children with SMA type 1, 2 and 3, aged between 1 and 18 were recruited between the 1st of January 2021 and the 30th of September 2021 from the neuropediatric follow-up consultations (FILNEMUS) that volunteered and from patient associations (AFM-Telethon et ECLAS). Parents received an information letter and were invited to contact Mrs Boursange (SB, researcher in charge of the interview) by telephone or by e-mail if they were interested. All participants signed written notices of no objection before participation.

The research consisted of 3 successive phases spaced out by a maximum of 1 month. The interviews took place by videoconference and were recorded:

1) **The parents completed a self-administrated in-house questionnaire** developed with the members of the Steering Comitee of the SMAPAR study (academics experts in clinical psychology, neuropediatricians and members of patient associations) and validated with members of patient association. Its objective was to describe the situation of the child and the family, the history of the illness, its management and the social inclusion of the parents and the child. The number of items varied according to the subject's answers, since an answer could lead to additional questions or, on the contrary, be limited to one answer. The time needed to complete the questionnaires was 45 minutes to 1 hour, and parents had the option of completing them in several rounds. It consisted of open- and closed-ended questions and visual analogic scales (VAS) that ranged from 0 (lowest agreement or satisfaction score) to 100 (highest agreement or satisfaction score). Dimensions explored were: general demographic questions

including gender, age, national, marital status, number of children, education level, socio-professional category, income, employment status, social supports and coverage, questions about the social and family environment, impact of the disease on the parents' overall psychological and physical health ; questions concerning the child and the SMA, the follow-up, the medical and therapeutic management, therapeutic decisions and effects observed following treatment, social inclusion of the child, questions concerning the organization of daily life and care of the child, support received by the parents and general questions on SMA and in particular on neonatal screening.

2) **A 1.5 to 2 hours in-depth semi-structured interview** conducted by a psychologist (SB) that consisted of 2 steps:

   i) Exploration of the parents' experience based on an open-ended question "Can you describe to me how you became the parent of « name of child »?" Other themes were proposed to the parents if they did not address them spontaneously: representation and role of the parent, relationship with the child, organization of the child's care, therapeutic decision-making, support needs, burden of the disease.

   ii) Exploration of parental representations using the "R" interview of Stern et al. (1). This is a semi-structured interview with 10 general themes and 28 questions. In this study, we selected five themes that were the most relevant to the objectives of the study: the description of the child and the parent as a parent, the emotions linked to the representations, the desires and fears regarding the future for the child and the parent, and the parent's self-esteem. The representations are evaluated in several ways: qualitatively, based on the parent's spontaneous verbal description and then, from a memory associated with the description, and quantitatively based on an VAS (0 – 100)

3) **7 validated self-questionnaires** to assess the middle-term psychological consequences in the parents'life.

   a. Depression: Beck Depression Inventory - II (BDI-II)

The Beck Depression Inventory - II (BDI-II) (2) is the most widely used tool for assessing depression. It is a self-questionnaire consisting of 21 items that assess the intensity of depression on a subjective level. The items are divided into two subscales, one evaluating the somatic aspects of depression and the other the psychological aspects. The subject estimates the frequency of symptoms during the last

two weeks, according to a 4-point Likert scale, corresponding to an increasing degree of intensity. The total score varies from 0 to 63. A score ≤11 indicates the absence of depression and a score≥ 12 indicates depression, qualified as mild for a score between 12 and 19, moderate for a score between 20 and 27, or severe for a score between 28 and 63.

b. Anxiety: State-Trait Anxiety Inventory (STAI-Y)

The State-Trait Anxiety Inventory - STAI-Y, translated into French by Bruchon-Schweitzer and Paulhan (3, 4). This scale is a self- questionnaire composed of 40 items divided into two subscales, the STAI-YA, which assesses state anxiety, and the STAI-YB, which assesses trait anxiety. Each item is rated on a 4-point Likert scale of "No," "Somewhat no," "Somewhat yes," and "Yes." This questionnaire differentiate trait anxiety, which is a stable personality characteristic, from state anxiety, which is related to a current situation of physical danger or psychological stress. Two scores are obtained, ranging from 0 to 80, by summing the items corresponding to each subscale, with a high score representing significant anxiety. According to the French version, a score < 45 corresponds to low anxiety, medium between 46 and 55, high between 56 and 65 and very high above 65.

c. Parental stress: Parental stress index short form (PSI-SF)

The level of parental stress illustrates an individual's ability to adapt to a situation. A high level of stress indicates a failure of coping strategies. We measured parental stress, with the short version of the Parental Stress Index (PSI-SF) (5). This self-questionnaire consisting of 36 statements has been used for several years in numerous studies (6). Parents respond according to a 5-point agreement scale. Three scales are calculated from this questionnaire: a) parental distress (PD), i.e., parenting malaise, b) dysfunction in the parent-child relationship, and c) perception of a difficult child. The internal consistency of the scale evaluated in a normal population ranges from 0.80 for the parent-child interaction dysfunction subscale to 0.91 for the total score and the test-retest reliability at 6 months (0.84) is also satisfactory (5).

d. Sense of coherence: sense of coherence self-questionnaire (SOC-13)

The sense of coherence is an indicator of resilience and a predictor of good health and well-being. It represents the capacity of a person to face stressfull events. The SOC-13 self-questionnaire is the short

version of the Sense of Coherence Questionnaire developed by Antonovsky (7) to evaluate the sense of coherence through 3 dimensions: comprehensibility (5 items), meaningfulness (4 items) and manageability (4 items) felt in daily life (7). The answers are given on a 7-point Likert scale, ranging from 1 (low SOC) to 7 (high SOC). The total score ranges from 13 to 91 and a high SOC score indicates a strong sense of coherence. The internal consistency of this questionnaire, evaluated in 127 studies, ranges from 0.70 and 0.90 (Cronbach's alpha) and the SOC scale is recognized as reliable, valid and applicable to multiple cultures (8).

e. Parental Burnout: Parental Burnout Assessment (PBA)

Parental burnout refers to a specific syndrome of exhaustion related to prolonged situations of emotional imbalance, where the parent's perceived stress and its impact exceeds the ressources available to cope (9). The Parental Burnout Assessment is a self-questionnaire developed from the testimonies of parents suffering from burnout (10). It consists of 23 items assessing the frequency with which parents experience symptoms of parental burnout on a 7-point Likert scale ranging from : (1) never to (7) every day. This questionnaire evaluates 4 dimensions (subscales): exhaustion in one’s parental role (9 items), contrast with previous parental self (difference in perception between the parents they were and those they have become - 6 items), role saturation (feeling of being fed up with one’s parental role - 5 items) and emotional distancing from one’s children (3 items). The internal consistency of this questionnaire ranges from 0.79 for emotional distancing, 0.90 for contrast with previous parental self and for role saturation, 0.93 for emotional exhaustion and 0.98 for the general consistency (10).

f. Marital support: Dyadic Adjustment Scale - DAS 16 (for parents in couple with the other parent)

Marital support, also known as marital satisfaction, will be measured using the revised Dyadic Adjustment Scale (DAS-16) (11). This scale is derived from the Dyadic Adjustment Scale (DAS), developed to assess the quality of dyadic adjustment in marriage and similar relationships (12). This scale is the most widely used tool for assessing marital adjustment and is increasingly used in contexts where couple adjustment is likely to be tested, such as in psycho-oncology, although its

multidimensional structure has been criticized (11). A revised form of the DAS-16 was proposed by Antoine et al. (11). It is composed of 16 items that are organized into two dimensions, "the degree of agreement in the couple" (DA) and "the quality of dyadic interactions" (IQ), which explain 52% of the total variance of the DAS scale. For each item, participants were asked to answer in reference to the previous month, using a Likert-type scale ranging from 2 to 7 points depending on the item. The psychometric qualities of this scale were evaluated with a sample of 123 couples. The internal consistency was satisfactory with Cronbach's coefficients ranging from 0.89 for the total and agreement scale and 0.75 for the quality of dyadic interaction scale. In addition, the DAS-16 is highly correlated with the DAS ($r = 0.97$; $p < 0.01$) and it discriminates distressed couples from those who are not (11).

g. The impact of the disease on family life: Family impact Childhood Disability questionnaire (FICD)

The French version of the Family Impact Childhood Disability (FICD) questionnaire was developed in Canada (13) and adapted in Europe by Guyard et al. (6). This self-questionnaire is specifically dedicated to children's disability situations. It consists of 20 items addressing positive and negative impact of the disability situation of the child in the following domains: time, social relationships, family activities, psychological consequences and financial expenses. For each item, responses are given on a 4-point Likert scale ranging from "not at all" to "to a significant degree". From these responses, 2 scores can be calculated: a global negative impact score (NFI) and a global positive impact score (PFI) between 10 and 40. The psychometric qualities of the FICD scale were evaluated in Canada in 2 studies : the first conducted with 87 families whose children aged about 5 years had a developmental disability (47% of the children had a multiple disability with developmental delay and hearing and/or visual problems, 24% of the children had a physical disability requiring constant assistance with daily care and 19% had Down's syndrome) (13) and the second conducted with 195 mothers of children aged 0-17 years with disabilities (55% of children had developmental disabilities, 6% motor disabilities, 19% mental health disabilities, 2% sensory disabilities, and 14% complex health problems) (14). Both subscales showed very good internal consistency and test-retest reliability. No respondent social desirability bias was found (15) and the FICD scale was shown to correlate with maternal depression ($r=.24$), parental stress ($r=.64$), and family adjustment ($r=.34$) (16).

The entire protocol was digitalized. The quantitaive data (self-administrated homemade questionnaire and 7 self-reported scales) was collected through the REDCAP interface, anonymized and hosted on a

secure server (17, 18). The in-depth interview was recorded, transcribed with pseudo-anonymization and transfered to the data analysis software NVivo 10 (version 1.6.1).

The study complies with the reference methodology MR-004. It received a favorable opinion from the Ethics Committee (N°: #00011928, December 15, 2020).

Bibliography

1. Stern, D.N., Robert-Tissot, C., Besson, G., Rusconi-Serpa, S., de Muralt, M., Cramer, B. & Palacio, F. « L'Entretien « R »: une méthode d'évaluation des représentations maternelles ». In: Lebovici, S., Mazet, P. & Visier, J.-P. (Eds), L'évaluation des interactions précoces entre le bébé et ses partenaires. Paris: Eshel (1989). p. 151-160.
2. Beck AT, Steer RA, Brown GK. Manual for the Beck depression inventory second edition (BDI-II). San Antonio: Psychological Corp. (1996)
3. Spielberger C.D. Manual for the State trait anxiety inventory. Palo Alto, CA: Consulting Psychologists Press. (1983)
4. Bruchon-Scweitzer M, Paulhan I. Manuel du STAI forme Y, adaptation française. Paris: ECPA. (1993)
5. Abidin, R. R. Parenting stress index Professional manual, (third edition). Lutz: Psychological Assessment Resources. (1995)
6. Guyard, A., Michelsen, S. I., Arnaud, C., Lyons, A., Cans, C., & Fauconnier, J. Measuring the concept of impact of childhood disability on parents: validation of a multidimensional measurement in a cerebral palsy population. Research in Developmental Disabilities (2012) 33(5):1594-1604. doi: 10.1016/j.ridd.2012.03.029
7. Antonovsky, A. "Personality and health: testing the sense of coherence model". In:Antonovsky, A. (dir), Friedman, S. (eds). Personality and Disease Howard New York: John Wiley and Sons (1990). p. 155-77
8. Eriksson, M. & Lindström, B. Validity of Antonovsky's sense of coherence scale: a systematic review. J Epidemiol Community Health (2005) 59(6):460–6. doi: 10.1136/jech.2003.018085
9. Hubert, S., & Aujoulat, I. Parental Burnout: When Exhausted Mothers Open Up. Frontiers in psychology, (2018) 9:1021. doi: 10.3389/fpsyg.2018.01021

10. Roskam, I., Brianda, M. E., & Mikolajczak, M. A Step Forward in the Conceptualization and Measurement of Parental Burnout: The Parental Burnout Assessment (PBA). Frontiers in psychology (2018) 9:758. doi :10.3389/fpsyg.2018.00758
11. Antoine, P., Christophe, V., & Nandrino, J.-L. Échelle d'ajustement dyadique : intérêts cliniques d'une révision et validation d'une version abrégée. L'Encéphale (2008) 34(1):38-46. doi:10.1016/j.encep.2006.12.005
12. Spanier, G.B. Measuring dyadic adjustment: new scales for assessing the quality of marriage and similar dyads. Journal of Marriage and the Family (1976) 38 :15-28. doi: 10.2307/350547
13. Trute, B., Hiebert-Murphy, D., & Levine, K. Parental appraisal of the family impact of childhood developmental disability: Times of sadness and times of joy. Journal of Intellectual & Developmental Disability (2007) 32(1): 1–9. doi: 10.1080/13668250601146753.
14. Trute, B., Benzies, K. M., Worthington, C., Reddon, J. R., & Moore, M. Accentuate the positive to mitigate the negative: mother psychological coping resources and family adjustment in childhood disability. Journal of Intellectual & Developmental Disability (2010) 35(1): 36–43. doi: 10.3109/13668250903496328
15. Benzies, K. M., Trute, B., Worthington, C., Reddon, J., Keown, L.-A., & Moore, M. Assessing psychological well-being in mothers of children with disability: evaluation of the parenting morale index and family impact of childhood disability scale. Journal of Pediatric Psychology (2011) 36(5) :506–516. doi:10.1093/jpepsy/jsq081.
16. Trute, B., & Hiebert-Murphy, D. Family adjustment to childhood developmental disability: a measure of parent appraisal of family impacts. Journal of pediatric psychology (2002) 27(3): 271–280. doi: 0.1093/jpepsy/27.3.271
17. Harris, P. A., Taylor, R., Thielke, R., Payne, J., Gonzalez, N., & Conde, J. G. (2009). Research electronic data capture (REDCap)--a metadata-driven methodology and workflow process for providing translational research informatics support. Journal of biomedical informatics. (2009) 42(2):377–381. doi: 10.1016/j.jbi.2008.08.010
18. Harris, P. A., Taylor, R., Minor, B. L., Elliott, V., Fernandez, M., O'Neal, L., McLeod, L., Delacqua, G., Delacqua, F., Kirby, J., Duda, S. N., & REDCap Consortium. The REDCap consortium: Building an international community of software platform partners. Journal of biomedical informatics. (2019) 95:103208. doi: 10.1016/j.jbi.2019.103208
